# Differential Relationships among Circulating Inflammatory and Immune Activation Biomediators and Impact of Aging and Human Immunodeficiency Virus Infection in a Cohort of Injection Drug Users

**DOI:** 10.3389/fimmu.2017.01343

**Published:** 2017-10-19

**Authors:** Gregory D. Kirk, Stewart Dandorf, Huifen Li, Yiyin Chen, Shruti H. Mehta, Damani A. Piggott, Joseph B. Margolick, Sean X. Leng

**Affiliations:** ^1^Department of Epidemiology, Johns Hopkins Bloomberg School of Public Health, Baltimore, MD, United States; ^2^Division of Infectious Diseases, Department of Medicine, Johns Hopkins University School of Medicine, Baltimore, MD, United States; ^3^Division of Geriatric Medicine and Gerontology, Johns Hopkins University School of Medicine, Baltimore, MD, United States; ^4^Department of Molecular Microbiology and Immunology, Johns Hopkins Bloomberg School of Public Health, Baltimore, MD, United States

**Keywords:** neopterin, sTNFR-1, sTNFR-2, IL-6, CRP, human immunodeficiency virus infection and aging

## Abstract

As individuals with human immunodeficiency virus (HIV) infection live longer, aging and age-related chronic conditions have become major health concerns for this vulnerable population. Substantial evidence suggests that chronic inflammation and immune activation contribute significantly to chronic conditions in people aging with or without HIV infection. As a result, increasing numbers of inflammation and immune activation biomediators have been measured. While very few studies describe their *in vivo* relationships, such studies can serve as an important and necessary initial step toward delineating the complex network of chronic inflammation and immune activation. In this study, we evaluated *in vivo* relationships between serum levels of neopterin, a biomediator of immune activation, and four commonly described inflammatory biomediators: soluble tumor necrosis factor (TNF)-α receptor (sTNFR)-1, sTNFR-2, interleukin (IL)-6, and C-reactive protein (CRP), as well as the impact of HIV infection and aging in the *A*IDS *L*inked to the *I*ntra*v*enous *E*xperience (ALIVE) study, a community-recruited observational study of former and current injection drug users (IDUs) with or at high risk for HIV infection in Baltimore, MD, USA. The study included 1,178 participants in total with 316 HIV-infected (HV+) and 862 HIV-uninfected (HIV−) IDUs. Multivariate regression analyses were employed, adjusting for age, sex, body mass index, smoking, hepatitis C virus co-infection, injection drug use, comorbidities, and HIV status (for all participants), and HIV viral load, CD4^+^ T-cell counts, and antiretroviral therapy (for HIV+ participants). The results showed significant impact of aging on all five biomediators and that of HIV infection on all but sTNFR-1. In the adjusted model, neopterin had positive associations with sTNFR-1 and sTNFR-2 (partial correlation coefficients: 0.269 and 0.422, respectively, for all participants; 0.292 and 0.354 for HIV+; and 0.262 and 0.435 for HIV−, all *p* < 0.0001). No significant associations between neopterin and IL-6 or CRP were identified. Such differential relationships between circulating neopterin and sTNFR-1, sTNFR-2, IL-6, and CRP may help inform their selection in future studies. These findings may also facilitate elucidation of underlying inflammatory and immune activation pathways that contribute to age-related chronic conditions, potentially leading to identification of key biomediators, particularly those upstream of CRP, as novel targets for intervention.

## Introduction

Human immunodeficiency virus (HIV) infection remains a major health problem worldwide. At present, approximately 34 million people are infected worldwide and there are about 50,000 new cases each year in the US alone. As effective treatment is available through combination antiretroviral therapy (cART) and new infections have increased among older adults, the number of older individuals living with HIV has risen dramatically over the past decade also. In fact, more than half of all HIV-infected persons in the US are now over the age of 50. Aging of the HIV-infected population is also evident in Asia and even in sub-Sahara Africa (1, 2). As HIV-infected (HIV+) individuals live longer, age-related chronic conditions commonly encountered in the geriatric population, some of which are also termed HIV-associated non-AIDS conditions, have become major health concerns for this vulnerable aging population despite suppression of HIV viral load to clinically undetectable levels by cART (3–6). One important example is frailty, a syndrome characterized by diminished physiologic reserve, increased vulnerability to stressors, and adverse health outcomes (3, 4). While much remains to be learned, it is believed that age-related senescent remodeling of the immune system, or immunosenescence, contributes significantly to the development of such chronic conditions and adverse health outcomes, and that HIV infection appears to accelerate immunosenescence (6–9).

Age-related chronic inflammation and dysregulated immune activation are considered cardinal features and likely mechanisms of immunosenescence. Biomediators commonly described in the literature include neopterin for immune activation and C-reactive protein (CRP), interleukin (IL)-6, soluble tumor necrosis factor (TNF)-α receptor (sTNFR)-1, and sTNFR-2 for chronic inflammation. In the geriatric population, we and others have shown that elevated serum levels of neopterin, CRP, IL-6, sTNFR-1, and sTNFR-2 are associated with frailty, disability, and mortality (10–13). In HIV infection, neopterin is shown to be elevated and its elevation is predictive of HIV disease progression (14–16). CRP, IL-6, and sTNFR-2 are also elevated with HIV infection and their elevated levels are associated with HIV disease progression and mortality in patients treated with cART (17–23). More recent studies in virologically suppressed HIV patients have shown persistent inflammation and immune activation, and that this persistent state of inflammation and immune activation is associated with aging, functional impairment, and AIDS defining events (24–29). In HIV+ men in the Multicenter AIDS Cohort Study (MACS), those with frailty had circulating CRP concentrations that were up to 50% higher than those in similar non-frail HIV+ men (30). However, *in vivo* relationships among these biomediators of chronic inflammation and immune activation have yet to be adequately investigated.

Neopterin, a GTP metabolite, is a well-established biomediator for immune activation primarily produced by monocytes and macrophages in response to stimulation with Th1-type cytokine interferon (IFN)-γ (31); its level increases with age (32) and in frailty (11). Both IL-6 and CRP are classic inflammatory biomediators and their elevated levels are considered the hallmark of age-related chronic inflammation, or “InflammAgeing” (33). TNF-α is a central player triggering inflammatory pathway or cascade as demonstrated in rheumatoid arthritis (34). TNF-α triggers inflammation through its two distinct but structurally homologous TNF receptors, the 55-kD receptor 1 (TNF-R1) and 75-kD receptor 2 (TNF-R2) (35, 36), and sTNFR-1 and sTNFR-2 are derived from TNF-R1 and TNF-R2, respectively, by proteolytic processing and have been shown to be reliable measurements for the *in vivo* activities of TNF-α. As such, sTNFR-1 and sTNFR-2 can be considered as more proximal inflammatory mediators than IL-6 and CRP. We have observed significant association between elevated levels of IL-6 and CRP among the *A*IDS *L*inked to the *I*ntra *V*enous *E*xperience (ALIVE) study participants, a large cohort of injection drug users (IDUs) with or at high risk for HIV infection (37). In the ALIVE study, we have also shown significant associations of serum levels of IL-6 and sTNFR-1 as well as an aggregate inflammatory index including IL-6 and sTNFR-1 levels with frailty and mortality (38). In addition, we have demonstrated significant *in vivo* associations between IL-6 and sTNFR-1 and sTNFR-2 levels (39). However, these are primarily biomediators of chronic inflammation. The objective of this study, therefore, was to further investigate the *in vivo* relationships between neopterin, a well-known biomediator of immune activation, and the above four inflammatory biomediators. This is built upon earlier work from Zangerle and colleagues who observed elevated neopterin and its association with sTNFR-1 in HIV-infected IDUs (40–42). We hypothesized that neopterin would have complex relationships with these inflammatory biomediators in which neopterin would be directly associated with sTNFR-1 and sTNFR-2 rather than IL-6 or CRP. As increasing numbers of immune activation and inflammatory biomediators have been evaluated in various settings, addressing this hypothesis is important in order to inform more accurate interpretation of existing data as well as their selection for evaluation in future studies. Delineating the *in vivo* relationships of these biomediators will advance our understanding of immune activation and inflammatory pathways as well as their role and interaction in contributing to chronic conditions and adverse health outcomes in the vulnerable aging population with HIV infection. To test this hypothesis, we conducted a cross-sectional analysis to evaluate the relationships between serum neopterin levels and levels of sTNFR-1, sTNFR-2, IL-6 and CRP in the ALIVE study, adjusting for age, sex, race, body mass index (BMI), cigarette smoking, comorbidities, hepatitis C infection, injection drug use, HIV status (for all participants), and HIV viral load, CD4 counts and cART (for HIV+ subgroup).

## Materials and Methods

### Study Population

The *A*IDS *L*inked to the *I*ntra *V*enous *E*xperience (ALIVE) study is a prospective cohort consisted of IDUs based in Baltimore, MD, USA. Methodology has been previously described (43). During semi-annual visits, ALIVE participants completed standardized questionnaires and submitted biospecimens for testing. Smoking and illicit injection drug use per participant were self-report of behaviors over the past 6 months. Frequency of injection drug use during the past 30 days prior to the visit was also recorded. Multimorbidity including diabetes mellitus, hypertension, obstructive lung disease, anemia, chronic kidney disease, and liver fibrosis were confirmed clinical diagnoses by medical history and records. HIV serology was determined using enzyme-linked immunosorbent assay (ELISA) with Western blot confirmation (Dupont, Wilmington, DE, USA). Hepatitis C infection was determined by a positive antibody titer using standard laboratory assay. For those who were HIV positive, CD4^+^ T cell counts and HIV viral load were measured routinely in a clinically certified laboratory. cART usage was self-reported by the participants and confirmed by medical or pharmacy record. Serum neopterin, sTNFR-1, sTNFR-2, IL-6, and CRP levels were measured as described below. A total of 1,190 participants had measurements of all 5 biomarkers at baseline. Residuals were examined and participants with high influence for one or more of the above biomediators were excluded (*n* = 10). Two participants were also removed due to incomplete data, leaving the sample size of 1,178 for this analysis. Johns Hopkins University Institutional Review Board approved the study and each participant provided written informed consent.

### Measurements of Serum Neopterin, sTNFR-1, sTNFR-2, IL-6, and CRP

Serum samples were obtained from each participant according to the standard protocol, and stored in aliquots at −80°C until analysis. Serum neopterin was measured using a commercially available competitive ELISA (ALPCO Diagnostics; Salem, NH, USA). The immunoassay has a sensitivity of 0.8 nM and an inter-assay coefficient of variance of 5.29%. Serum sTNFR-1, sTNFR-2, IL-6, and CRP were measured using commercially available ELISA according to the procedures provided by manufacturers. Serum sTNFR-1 and sTNFR-2 were measured using DuoSet ELISA kits (R&D Systems, Minneapolis, MN, USA) with a sensitivity of 12.5 or 7.8 pg/ml and an inter-assay CV of 4.9 or 6.1%, respectively. Serum IL-6 and CRP were measured using High-Sensitivity Quantikine kits (R&D Systems) with detection ranges of 0.156–10.0 and 31.25–2,000 pg/mL and inter-assay coefficients of variance of 5.7 and 6.4%, respectively. Measurements were performed in duplicate and repeated if the measures differed by more than 15% or were out of the measureable range. The average of the two values in duplicate was used for analyses.

### Statistical Analysis

Frequency distributions were determined for baseline population characteristics. Fisher’s exact test and Student’s *t*-test were used to determine differences between categorical and continuous data by HIV status, respectively. Medians and Interquartile Range (IQR) were calculated for all five biomediators and the non-parametric Wilcoxon–Mann–Whitney test was used to compare distributions between groups stratified by HIV status. Bivariate associations between neopterin and each of the inflammatory biomediators (sTNFR-1, sTNFR-2, IL-6, and CRP) were evaluated using cross-tabulations of means and SDs of sTNFR-1, sTNFR-2, IL-6, and CRP levels by quartiles of neopterin; analysis of variance was used to compare group differences. Multiple linear regression was used to examine the relationships between neopterin and each of the inflammatory biomediators (sTNFR-1, sTNFR-2, IL-6, and CRP). Separate analyses were performed for each pairs of biomediators adjusting for age, sex, race, BMI, cigarette smoking, number of comorbidities (0 or 1, 2, ≥3), hepatitis C infection, number of injection drug use within the past 30 days, HIV status (for all participants), and HIV viral load, CD4 counts and cART (for HIV+ group). To account for non-normal distributions, levels of neopterin, sTNFR-1, sTNFR-2, IL-6, and CRP were log transformed to approximate normality for linear regression analyses. Regression effects can be interpreted as a one percent change in the median covariate value for each unit change in the median outcome value. Multi-collinearity was examined using variance inflation factors with 2.5 as a cutoff. Assumptions were checked for all models by examining error properties and residual plots. All analyses were performed using SAS statistical software (Version 9.2, Cary, NC, USA).

## Results

Out of 1,178 participants (all participants) included in this analysis, 316 were HIV+ and 862 were HIV− participants, giving a 26.8% prevalence of HIV infection. The majority of the study participants were African-American (87.5%) and male (64.9%) with a mean age of 46.8 (range 21.2–78.1) years. Daily injection drug use was reported in 21.1% of the population. Table 1 summarizes baseline demographic and clinical characteristics as well as medians (IQR) of neopterin, sTNFR-1, sTNFR-2, IL-6, and CRP levels of all participants as well as HIV+ and HIV− study groups. Compared to HIV− participants, those who were HIV+ were more likely to be African-American, never married, unemployed, use injection drugs less often, inject fewer times within the past 30 days, consume fewer alcoholic drinks/day, and have two or more comorbidities. The prevalence of hepatitis C infection was 86% among all participants included in this analysis with hepatitis C co-infection present in all (100%) HIV+ participants and 80.7% in HIV− participants (*p* = 0.0001). There was no statistical difference with regard to age, sex, smoking, or BMI between HIV+ and HIV− groups. For HIV+ participants, median (IQR) CD4^+^ T-cell count and HIV viral load were 304 cells/mm^3^ (180–437) and 961 copies/ml (400–28,000), respectively. Among them, 163 were treated with cART with majority being treated with protease inhibitors (57.7%) or non-nucleotide reverse transcriptase inhibitors (18.5) alone and the remaining 148 (46.8%) had no cART. Median (IQR) levels of immune activation and inflammatory biomediator for all participants were 16.52 nmol/ml (10.85–26.78) for neopterin, 1,491 pg/ml (1,260–1,834) for sTNFR-1, 4,976 pg/ml (3,833–6,900) for sTNFR-2, 1.61 pg/ml (1.01–2.75) for IL-6, and 1,566 pg/ml (546–4,632) for CRP (Table 1). HIV+ participants had significantly higher neopterin, IL-6, and sTNFR-2 levels than HIV− participants (median 25.98 vs 14.36, *p* < 0.0001, 1.81 vs 1.50 pg/mL, *p* = 0.0001 and 9,682 vs 4,487 pg/mL, *p* < 0.0001, respectively), while there was no significant difference in sTNFR-1 or CRP levels between the two study groups (1,484 vs 1,492 pg/mL, *p* = 0.416 and 1,307 vs 1,723, *p* = 0.069, respectively). The median neopterin levels in HIV− IDUs were higher than that typically seen in other HIV− populations, likely due to drug use or other infections.

**Table 1:** Characteristics of ALIVE study participants: all participants and stratified by human immunodeficiency virus (HIV) status.

	All participants (*n* = 1,178)	HIV positive (*n* = 316)	HIV negative (*n* = 862)	*p*-Value
Age, mean in years (95% CI)	46.8 (46.3–47.2)	46.9 (46.2–47.6)	46.7 (46.1–47.3)	0.762
**Sex**				
Male	765 (64.9)	203 (64.2)	562 (65.2)	0.783
Female	413 (35.1)	113 (35.8)	300 (34.8)	

**Race**				
White/other	147 (12.5)	20 (6.3)	127 (14.7)	0.0001
African-American	1,031 (87.5)	296 (93.7)	735 (85.3)	

**Marital status**				
Never married	776 (65.9)	230 (73.3)	546 (63.4)	0.002
Ever married	399 (33.9)	84 (26.8)	315 (36.6)	

**Employed**				
No	870 (73.9)	253 (80.1)	617 (71.7)	0.004
Yes	306 (26.0)	63 (19.9)	243 (28.3)	

**Cigarette smoker**				
No	181 (15.4)	56 (17.8)	125 (14.5)	0.164
Yes	994 (84.4)	258 (82.2)	736 (85.5)	

**IV drug use in past 6 months**				
None	637 (54.1)	182 (57.6)	455 (52.8)	0.007
<Daily	293 (24.9)	87 (27.5)	206 (23.9)	
≥Daily	248 (21.1)	47 (14.9)	201 (23.3)	
Number of injections in past 30 days, median (IQR)	25 (4–60)	12 (4–35)	30 (4–60)	0.038

**Number of alcoholic drinks/day**				
0	549 (46.6)	173 (54.8)	376 (43.6)	0.009
1–2	339 (28.8)	78 (24.7)	261 (30.3)	
3–4	166 (14.1)	38 (12.0)	128 (14.9)	
≥5	124 (10.5)	27 (8.5)	97 (11.3)	

**Comorbidities**				
0	293 (24.9)	43 (13.6)	250 (29.0)	<0.0001
1	406 (34.5)	87 (27.5)	319 (37.0)	
2	294 (25.0)	105 (33.2)	189 (21.9)	
≥3	185 (15.7)	81 (25.6)	104 (12.1)	

**Body mass index**				
<30	931 (79.0)	259 (82.0)	672 (78.0)	0.135
≥30	247 (21.0)	57 (18.0)	190 (22.0)	

**Hepatitis C infection**				
No	166 (14.0)	0 (0.0)	166 (19.3)	<0.0001
Yes	1,012 (86.0)	316 (100.0)	696 (80.7)	
CD4^+^ T-cells, cells/mm^3^, median (IQR)		304 (180–437)		
HIV viral load, copies/ml, median (IQR)		961 (400–28,000)		

**cART use in past 6 months**				
No		148 (46.8)		
Yes		163 (51.6)		
Neopterin, nmol/ml, median (IQR)	16.52 (10.85–26.78)	25.98 (16.49–39.08)	14.36 (9.83–21.88)	<0.0001
Factor (TNF)-α receptor (sTNFR)-1, pg/ml, median (IQR)	1,491 (1,260–1,834)	1,484 (1,257–1,955)	1,492 (1,261–1,808)	0.416
sTNFR-2, pg/ml, median (IQR)	4,976 (3,833–6,900)	9,682 (5,113–9,213)	4,487 (3,592–5,964)	<0.0001
IL6, pg/ml, median (IQR)	1.61 (1.01–2.75)	1.81 (1.21–3.12)	1.50 (0.95–2.67)	0.0001
C-reactive protein, pg/ml, median (IQR)	1,566 (546–4,632)	1,307 (498–3,815)	1,723 (552–4,878)	0.069

*Values are number (%) unless otherwise noted*.

*cART, combination antiretroviral therapy; IQR, interquartile range*.

Potential associations of age, sex, race, and HIV status in all participants were assessed by bivariate regression analyses (Table 2). Among all participants, neopterin and IL-6 levels were significantly associated with age, sex, and HIV status, but not race. Levels of sTNFR-1 were significantly associated with age and race but not sex or HIV status. Levels of sTNFR-2 were significantly associated with age and HIV status but not sex or race. CRP levels were significantly associated with age, sex, race, and HIV status. Median neopterin levels were increased by 68.2%, IL-6 levels were increased by 19.8%, and sTNFR-2 levels by 51.5% in HIV+ participants compared to HIV− participants (all *p* < 0.05). Females had median IL-6 and CRP levels that were higher compared to males by 17.7 and 33.0%, respectively, both *p* < 0.05. African-Americans had median sTNFR-1 and CRP levels that were lower compared to all other races by 10 and 34.4%, respectively, both *p* < 0.05.

**Table 2:** Effects of age, sex, race, and human immunodeficiency virus (HIV) status on circulating levels of neopterin, sTNFR-1, Factor (TNF)-α receptor (sTNFR)-2, IL-6, and C-reactive protein (CRP) in ALIVE study participants as shown by regression coefficients (95% CI).^a^

	Age (years)		Female vs male		African-American vs White/other		HIV positive vs HIV negative	
	Coefficient	95% CI	Coefficient	95% CI	Coefficient	95% CI	Coefficient	95% CI
Neopterin (nmol/ml)	1.006*	(1.001–1.01)	0.893*	(0.828–0.963)	1.025	(0.919–1.144)	1.682*	(1.559–1.815)
sTNFR-1 (pg/ml)	1.006*	(1.004, 1.008)	1.006	(0.969, 1.045)	0.902*	(0.854, 0.953)	1.017	(0.977, 1.060)
sTNFR-2 (pg/ml)	1.005*	(1.002, 1.008)	1.018	(0.964, 1.076)	0.945	(0.872, 1.023)	1.515*	(1.435, 1.600)
IL-6 (pg/ml)	1.008*	(1.001–1.015)	1.177*	(1.053–1.315)	1.071	(0.912–1.257)	1.198*	(1.063–1.350)
CRP (pg/ml)	0.983*	(0.972–0.994)	1.330*	(1.104–1.601)	0.656*	(0.501–0.858)	0.804*	(0.658–0.983)

*p < 0.05.

*^a^Since log-transformed scores were used in the regression analyses, values presented are exponentiated regression coefficients*.

The relationships between levels of neopterin and those of sTNFR-1, sTNFR-2, IL-6, CRP in all participants were evaluated next. First, the means and SDs of sTNFR-1, sTNFR-2, IL-6, and CRP levels were cross-tabulated across quartiles of neopterin levels. We found a stepwise increase in sTNFR-1 and sTNFR-2 levels across neopterin quartiles (Table 3). There were no significant differences across neopterin quartiles for IL-6 and CRP.

**Table 3:** Mean (SD) of sTNFR-1, factor (TNF)-α receptor (sTNFR)-2, IL-6, and C-reactive protein (CRP) levels across neopterin quartiles.

Neopterin quartiles (range in nmol/ml)	0–25% (0–10.88)	26–50% (10.92–16.49)	51–75% (16.51–27.10)	76–100% (27.13–174.04)	*p*-Value*
sTNFR-1 (pg/ml)	1,500 (584)	1,522 (479)	1,641 (566)	1,899 (875)	<0.0001
sTNFR-2 (pg/ml)	4,406 (1,658)	4,867 (2,102)	6,067 (2,921)	8,522 (4,754)	<0.0001
IL-6 (pg/ml)	3.84 (8.92)	3.31 (6.40)	3.58 (9.17)	3.07 (5.89)	0.776
CRP (pg/ml)	3,491 (4,521)	3,539 (4,647)	3,999 (7,879)	3,246 (5,765)	0.444

*p-Value is from the trend test of the differences in sTNFR-1, sTNFR-2, IL-6, and CRP Levels across Neopterin quartiles.

**Figure 1:**
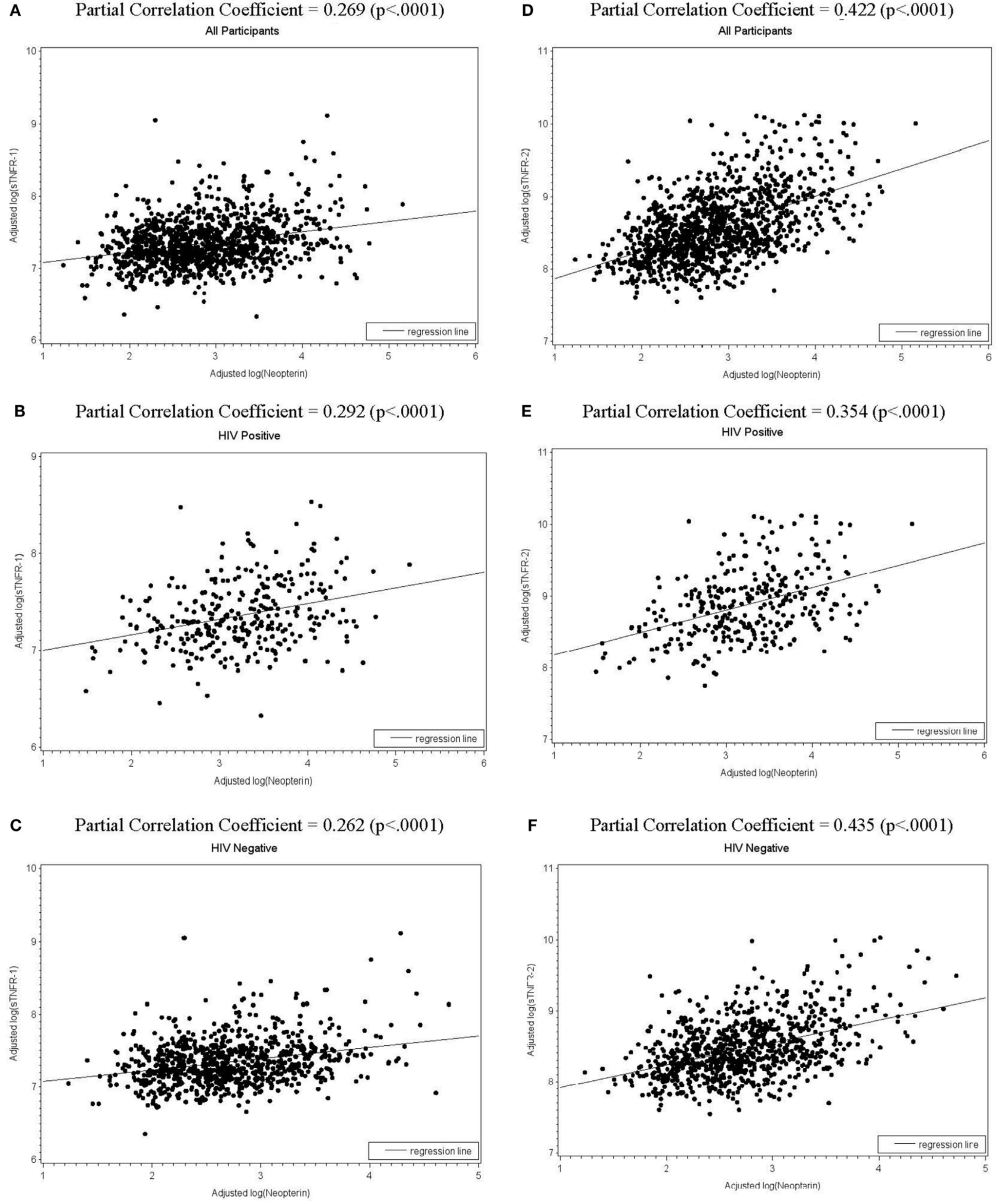
Scatterplots of total (all participants), human immunodeficiency virus (HIV)-positive, and HIV-negative populations with regression line shows, respectively, the fitted relationships of log(neopterin) with log(sTNFR-1)**(A–C)** and with log(sTNFR-2) **(D–F)**, adjusting for age, sex, race, number of comorbidities (0 or 1, 2, 3 or more), cigarette smoking, hepatitis C infection, body mass index, and number of injections in the past 30 days. CD4^+^ T-cell counts, HIV viral load, and combination antiretroviral therapy (cART) use in past 6 months were also adjusted for HIV-positive subgroup.

We then assessed the associations between log-transformed levels of neopterin and those of sTNFR-1, sTNFR-2, IL-6, and CRP in all participants as well as HIV+ and HIV− participants, adjusting for age, sex, race, BMI, cigarette smoking, number of comorbidities, hepatitis C infection, number of injections in the past 30 days, HIV status (for all participants), as well as CD4^+^ T-cell counts, HIV viral load, and cART (for HIV+ group only). log(neopterin) and log(sTNFR-1) were associated with each other in all participants as well as in HIV+ and HIV− groups (partial correlation coefficient *r* = 0.269, 0.292, and *r* = 0.262, respectively, all *p* < 0.0001, Figures 1A–C). Similarly, log(neopterin) and log(sTNFR-2) were associated with each other for all participants, HIV+, and HIV− groups (*r* = 0.422, 0.354, and *r* = 0.435, respectively, all *p* < 0.0001, Figures 1D–F). log(neopterin) had minimal or insignificant associations with log(CRP) or log(IL-6) in all population, HIV+, and HIV groups (data not shown).

Multiple linear regression analyses were performed using log(neopterin) as predictor and log(sTNFR-1), log(sTNFR-2), log(IL-6), and log(CRP) as outcome measures for all participants, HIV+, and HIV− groups, adjusting for age, sex, race, BMI, cigarette smoking, number of comorbidities, hepatitis C infection, and number of injection drug use in the past 30 days. Analyses for all participants were also adjusted for HIV status and those for the HIV+ group were also adjusted for HIV viral load, CD4^+^ T-cell counts and cART (Table 4). The results indicate that log(neopterin) had significantly positive associations with log(sTNFR-1) and log(sTNFR-2) for all participants [regression coefficients 0.467 (SE, 0.054) and 0.569 (0.039), respectively, both *p* < 0.0001] as well as for both HIV+ participants [0.542 (0.104) and 0.476 (0.073), both *p* < 0.0001, respectively] and HIV− participants [0.487 (0.061) and 0.622 (0.044), both *p* < 0.0001, respectively]. On the other hand, log(neopterin) had no significant associations or negligible regression coefficients (likely spuriously negative) with log(CRP) or log(IL-6) in all participants, HIV+ or HIV− participants.

**Table 4:** Adjusted regression coefficients sTNFR-1, factor (TNF)-α receptor (sTNFR)-2, IL-6, or C-reactive protein (CRP) (SE).

		All participants	Human immunodeficiency virus (HIV) positive	HIV negative
log (Neopterin)	log (sTNFR-1)	0.467 (0.054)**	0.542 (0.104)**	0.487 (0.061)**
	log (sTNFR-2)	0.569 (0.039)**	0.476 (0.073)**	0.622 (0.044)**
	log (IL-6)	−0.042 (0.018)*	−0.034 (0.042)	−0.040 (0.020)*
	log(CRP)	−0.014 (0.011)	−0.030 (0.021)	−0.003 (0.013)

**p < 0.0001, *p < 0.05.

*All models adjusted for age, sex, race, number of comorbidities (0 or 1, 2, ≥3), cigarette smoking, Hepatitis C infection, body mass index, and number of injections in the past 30 days*.

*HIV status was adjusted for all participants*.

*CD4^+^ T-cell counts, HIV viral load, and cART use in past 6 months were adjusted for HIV-positive subgroup*.

## Discussion

In this study, we have observed, for the first time, differential *in vivo* associations between circulating neopterin and four commonly tested inflammatory biomediators (sTNFR-1, sTNFR-2, IL-6, and CRP) with significant impact of HIV infection and aging in a cohort of IDUs with and at risk for HIV infection.

As the role of chronic inflammation and immune activation in HIV disease progression as well as in the manifestations of aging and age-related chronic conditions has become widely recognized, increasing number of biomediators (or biomarkers by some) of chronic inflammation and immune activation have been evaluated. The challenge is then to appropriately interpret the results and gain biologically informed insights from their evaluation. This study serves as an important initial step toward addressing this challenge through evaluating *in vivo* relationships between neopterin and four commonly tested inflammatory mediators in the ALIVE study. Theoretically, since immune activation leads to inflammation, neopterin would have significant associations with all four inflammatory biomediators. Our findings, however, demonstrate significant and positive associations of neopterin with sTNFR-1 and sTNFR-2 only, not with IL-6 or CRP, suggesting more complex *in vivo* relationships. One possible explanation for such preferential associations is that monocytes and macrophages are a main source of neopterin, sTNFR-1 and sTNFR-2 production. However, IL-6 is also considered as a monokine and no consistent association was observed between neopterin and IL-6 levels. Another biological plausible explanation is that neopterin, being a biomediator of immune activation is associated with more proximal inflammatory biomediators (sTNFR-1 and sTNFR-2) rather than distal biomediators (IL-6 and CRP). It is conceivable that the levels of IL-6 and CRP, the two classic and yet more distal inflammatory biomediators, are regulated by various factors and local milieu in addition to the underlying immune activation. This is illustrated by the result that CRP levels were lower in HIV+ participants compared to HIV− individuals with the difference reaching borderline statistical significance (*p* = 0.069, Table 1). This is because CRP is mainly produced by the liver (44) and hepatitis C infection, which was positive in all HIV+ study participants, could lead to significant liver damage. In fact, our previous study observed lower CRP levels in the ALIVE participants with HIV and hepatitis C virus co-infections (37). Alternatively, the observed associations of neopterin with sTNFR-1 and sTNFR-2 may be secondary to a Th1-type response as elevated neopterin and soluble TNF-α receptors are, at least under certain conditions, downstream products of IFN-γ activation (45), while IL-6 and CRP are typically considered as markers for Th2-type response.

The stronger association of neopterin with sTNFR-2 than with sTNFR-1 is not surprising but worthwhile emphasizing. sTNFR-2 is primarily produced by immune cells, particularly CD8^+^ T cells which could be activated by residual HIV infection and significantly impacted by immunosenescence (46, 47). In addition, sTNFR-2 rather than sTNFR-1 has shown consistent association with HIV infection and disease progression (48–50). Our findings confirmed the earlier work from from Zangerle et al. cited above (42) in a much larger sample and further expanded to include other inflammatory mediators. Stein and colleagues evaluated levels of sTNFR-2, neopterin, HIV RNA, and β_2_-microglobulin levels in the MACS and reported that high baseline sTNFR-2 levels were predictive for HIV disease progression or death (23). However, that study was conducted only in men with early-stage of HIV infection and did not include sTNFR-1, IL-6, or CRP levels. Whether sTNFR-2 has important regulatory function to activate or accelerate inflammatory pathways in response to neopterin beyond TNF-α cascade in the setting of HIV infection and aging and, therefore, represents a potential interventional target upstream of CRP and IL-6, deserve further investigation. This therapeutic implication has been demonstrated in the field of cardiovascular disease as serum CRP measurement is now incorporated in routine clinical assessment of chronic inflammation and atherosclerosis and studies have started to move upstream to identify novel target for vascular protection (51).

A major strength of this study is the availability of data for all five biomediators measured in the same blood sample collected at the same visit from the same individual in a large cohort study. It makes this analysis feasible and the observed *in vivo* associations biologically meaningful. Additional strengths include that standard or high sensitive ELISA assays were employed as appropriate for measuring these mediators, avoiding pitfalls associated with multiplex assays [reviewed in Ref. (52)]. This study also has several limitations. First, this is a cross-sectional analysis. We could not determine causal directionality of the identified associations. We have adjusted for a number of potential covariates commonly known for IDUs (such as IV drug use, injection frequency, and hepatitis C infection) and HIV infection (viral load, CD4 counts, and cART therapy) as well as comorbidities. We have also vigorously excluded participants with outlier values. However, other potential confounding factors that were not in the dataset or unknown to the participants could not be completely eliminated. In addition, we only included neopterin, sTNFR-1, sTNFR-2, IL-6, and CRP in this study. Other immune and inflammatory mediators are also likely important in the setting of HIV infection and aging. Finally, the ALIVE study cohort is a rather unique population identified by the IDU behavior. Results from this study will need to be confirmed in other HIV+ and HIV− populations. Despite these limitations, findings from this study do support our original hypothesis and suggest significant and positive *in vivo* associations of immune activation biomediator neotperin with proximal inflammation biomediator, sTNFR-1 and sTNFR-2 rather than more distal ones, IL-6 and CRP. These findings, if confirmed and further expanded, may facilitate elucidation of underlying inflammatory and immune activation pathways that contribute to the development of age-related chronic conditions as well as the impact of HIV infection, aging, and immunosenescence. They may also help not only inform their selection of for evaluation in the future studies, but also promote further investigations into their role and regulation, particularly those upstream of CRP as novel interventional targets for frailty and other chronic conditions in older adults with or without HIV infection.

## Ethics Statement

This study was carried out in accordance with the recommendations of human research subjects, the Johns Hopkins Institution Review Board (IRB)’s with written informed consent from all subjects. All subjects gave written informed consent in accordance with the Declaration of Helsinki. The protocol was approved by the Johns Hopkins IRB.

## Author Contributions

GK, SM, DP, JM, and SL contributed to overall design and data interpretation of this study. SD contributed to data analyses. HL and YC contributed to biomediator measurements. GK and SM are lead investigators for the ALIVE study. All authors contributed to manuscript writing and editing.

## Conflict of Interest Statement

The authors declare that the research was conducted in the absence of any commercial or financial relationships that could be construed as a potential conflict of interest. The reviewer DG and handling editor declared their shared affiliation.
